# Fatigue in pediatric patients with inflammatory bowel disease: a multicenter study by the SEGHNP

**DOI:** 10.1093/crocol/otag005

**Published:** 2026-02-13

**Authors:** Rafael Martin-Masot, Marta Velasco Rodríguez-Belvís, Gemma Pujol Muncunill, Laura Palomino, César Sánchez Sánchez, Javier Martín de Carpi, Víctor Manuel Navas-López, Inflammatory Bowel Disease Working Group of SEGHNP (Spanish Pediatric Gastroenterology, Hepatology and Nutrition Society)

**Affiliations:** Pediatric Gastroenterology and Nutrition Section, Regional University Hospital of Málaga, Málaga, Spain; Departamento de Farmacología y Pediatría, Facultad de Medicina, Universidad de Málaga, 29071 Málaga, Spain; Pediatric Gastroenterology and Nutrition Section, Niño Jesús University Children’s Hospital, Madrid, 28009, Spain; Pediatric Gastroenterology, Hepatology and Nutrition Service, Sant Joan de Déu Hospital, Esplugues de Llobregat, Barcelona, 08950, Spain; Pediatric Gastroenterology and Nutrition Section, Niño Jesús University Children’s Hospital, Madrid, 28009, Spain; Pediatric Gastroenterology Section, General University Hospital Gregorio Marañón, Madrid, 28007, Spain; Pediatric Gastroenterology, Hepatology and Nutrition Service, Sant Joan de Déu Hospital, Esplugues de Llobregat, Barcelona, 08950, Spain; Pediatric Gastroenterology and Nutrition Section, Regional University Hospital of Málaga, Málaga, Spain

**Keywords:** children, pediatric inflammatory bowel disease, fatigue, health-related quality of life, IMPACT-III questionnaire

## Abstract

**Background:**

Fatigue is a common and disabling symptom in pediatric inflammatory bowel disease (IBD), often persisting even during clinical remission and reflecting a multifactorial origin. Despite its significant impact on patients' lives, it remains under-recognized. The IMPACT-III and IMPACT-III-P questionnaires facilitate fatigue assessment within a biopsychosocial framework of health-related quality of life (HRQOL).

**Methods:**

In this multicenter study supported by the Spanish Society of Pediatric Gastroenterology, Hepatology and Nutrition (SEGHNP), 382 patients aged 10-17 years and their caregivers from 37 hospitals completed the IMPACT-III and IMPACT-III-P questionnaires between February 2021 and June 2023. Fatigue-related items were analyzed, and predictive models were developed using univariate and multivariate logistic regression.

**Results:**

A total of 370 patient questionnaires were included in the analysis. The median age at diagnosis was 11.3 years (interquartile range [IQR] 8.7-13.3), and at assessment, 14.4 years (IQR 12.4–16.1). Males represented 56% of the cohort, and 61.1% had Crohn’s disease. Treatments included immunosuppressants (44.6%), 5-ASA (33.7%), biologics (30.8%), corticosteroids (6%), and other therapies (27.8%). Fatigue was reported by 81.1% of patients, including 77.5% of those in clinical remission. Severe fatigue was significantly associated with female sex, older age, active disease, and dietary treatment. Conversely, absence of fatigue was independently associated with male sex, earlier pubertal stage, and not receiving biologics. Notable discrepancies were observed between patient and caregiver perceptions of energy levels. Fatigue correlated with significantly lower HRQOL scores across all IMPACT-III domains. In Crohn’s disease, the strongest impacts were observed in the social and systemic domains, whereas in ulcerative colitis, emotional and physical domains were more affected. Patients without severe fatigue consistently scored higher in all domains.

**Conclusion:**

Fatigue is a highly prevalent and multifactorial symptom in pediatric IBD, with a marked negative impact on quality of life, even in clinical remission. The IMPACT-III and IMPACT-III-P questionnaires are valuable tools for its assessment and highlight the need for routine, systematic evaluation of fatigue to guide holistic and individualized management strategies.

Key Messages

- **What is already known?** 
  - Fatigue is a common and disabling symptom in pediatric inflammatory bowel disease (IBD), often persisting despite clinical remission and negatively affecting quality of life.
- **What is new here?** 
  - This is the largest multicenter study using the validated Spanish versions of the IMPACT-III and IMPACT-III-P questionnaires to explore fatigue prevalence, predictors, and its impact on quality of life in pediatric IBD.
- **How can this study help patient care?** 
  - Systematic assessment of fatigue using standardized tools can identify high-risk patients and guide more personalized, holistic care strategies in pediatric IBD.

## Background

Fatigue is defined as a persistent and subjective sensation of tiredness, characterized by diminished physical and mental capacity, which is not alleviated by rest or prolonged sleep.^1^ Is one of the most frequent and burdensome symptoms in children and adolescents with inflammatory bowel disease (IBD), affecting multiple aspects of daily life including physical activity, concentration, emotional stability, and social participation.^2^ Notably, fatigue often persists even during clinical remission, suggesting it is not merely a marker of active inflammation but a complex, multifactorial phenomenon. Despite its clinical relevance, fatigue is rarely assessed systematically in pediatric IBD, and there is no disease-specific tool currently available to capture it comprehensively in this population. In this context, health-related quality of life (HRQOL) instruments can serve as surrogate tools to evaluate fatigue in a broader biopsychosocial framework.

The IMPACT-III questionnaire is the most widely used HRQOL tool specifically designed for pediatric IBD and includes 35 items answered by patients aged 10-17 years.^3^ Although it was originally structured around 6 conceptual domains—bowel and systemic symptoms, emotional and social functioning, body image, and treatment concerns—a more recent psychometric analysis proposed a refined model that reorganizes the items into 4 robust domains: general well-being, emotional functioning, social functioning, and body image. This restructuring improves internal consistency and interpretability without removing any items, thus preserving key content.^4^ Of relevance to fatigue, items 6 and 32 directly explore perceived energy and tiredness levels, offering a useful, albeit indirect, means to monitor fatigue. Questions 6 and 32 fall under the “systemic symptoms” domain in the 6-domain version of the IMPACT-III, and under the “well-being” domain in the 4-domain version. In the present study, we employed the recently developed and psychometrically validated Spanish versions of the IMPACT-III and its parent-proxy version (IMPACT-III-P), which demonstrated excellent reliability and validity in a large multicenter cohort from the Spanish Society of Pediatric Gastroenterology, Hepatology and Nutrition (SEGHNP). These tools offer a culturally appropriate and psychometrically sound approach to exploring HRQOL—including fatigue—in Spanish pediatric IBD populations. The aims of the study were to assess the prevalence of fatigue in a cohort of pediatric patients with IBD, the factors associated with it, and how it affected the other domains of the IMPACT-III.

## Methods

Both IMPACT-III and IMPACT-III-P are self-administered questionnaires, for children from 10 to 17 years old, that include 35 items, answered with a 5-point Likert scale. They evaluate 6 domains: bowel symptoms, systemic symptoms, emotional functioning, social functioning, body image and treatment or interventions. Higher scores indicate better HRQoL. We used the questionnaires validated in previous studies by our group.^5^ Members of the Spanish Society of Pediatric Gastroenterology, Hepatology and Nutrition (SEGHNP) were invited to recruit p-IBD patients from 10 to 17 years old (inclusive) and their parents/caregivers to complete the consensus versions of both questionnaires, from February 2021 to June 2023. We calculated the average of responses to items Q6 and Q32, following the general approach used in the IMPACT-III scoring system. For descriptive purposes, we considered mean scores >75 to indicate absence of fatigue; scores ≤75 as suggestive of at least mild fatigue; ≤50 as moderate fatigue; and ≤25 as severe fatigue. These thresholds have not been formally validated and are used here solely as an exploratory framework, in alignment with the methodology outlined by Turner et al.^6^ Predictive models were constructed using univariate and multivariate logistic regression analyses. All available variables were initially assessed in the univariate analysis, and those showing statistically significant differences or a trend (*P* < 0.15) were subsequently included in the multivariate analysis. In addition, variables theoretically or empirically considered relevant to the dependent variable were also included in the final model. The strength of the association between each predictive variable and the outcome was estimated using odds ratios (ORs) with corresponding 95% confidence intervals (CIs). Only data related to severe fatigue and absence of fatigue are presented, as exploratory analysis did not yield conclusive results for intermediate fatigue categories.

The study data were collected and managed using the REDCap electronic data capture tools hosted by the SEGHNP (www.seghnp.org). Technical support was provided by the AEGREDCap Support Unit, shared with the Spanish Association of Gastroenterology (AEG). REDCap (Research Electronic Data Capture) is a secure, web-based application designed to support data capture for research studies, providing (1) an intuitive interface for validated data entry; (2) audit trails for tracking data manipulation and export procedures; (3) automated export procedures for seamless data downloads to common statistical packages; and (4) procedures for importing data from external sources. Data were analyzed using the statistical package SPSS, release 20.0 for Windows (SPSS, Inc.). Figures were created using Prism 10 for macOS, version 10.4.2 (534), dated March 29, 2025. Questionnaires with missing responses were excluded from the analysis.

The study was approved by the Institutional Review Boards of all collaborating centers and was performed in full accordance with the Declaration of Helsinki. All participating parents/caregivers and patients over 12 years old signed a written informed consent.

## Results

We recruited up to 382 families from 37 hospitals. A total of 370 patients' IMPACT-III questionnaires and 361 parents/caregivers' IMPACT-III questionnaires were included. Five questionnaires from children (1.3% of 375) and 17 from parents (4.5% of 378) were removed due to incomplete data. The sample characteristics are summarized in Table 1.

**Table 1:** Demographical and medical characteristics of PIBD patients (*n* = 370).

Male sex, *n* (%)	207 (56)
**Age at diagnosis, years, median (IQR)**	11.3 (8.7-13.3)
**Age at assessment, years, median (IQR)**	14.4 (12.4-16.1)
**Type of IBD *n* (%)**	
**CD**	226 (61.1)
**UC**	128 (34.6)
**IBD-U**	16 (4.3)
**Paris classification UC+ IBD-U, *n* (%)**	144 (38.9)
**E1: Ulcerative proctitis**	11 (8)
**E2: Left-sided UC (distal to splenic flexure)**	27 (19)
**E3: Extensive (hepatic flexure distally)**	10 (7)
**E4: Pancolitis (proximal to hepatic flexure)**	95 (66)
**Severity (severe defined by PUCAI** **≥** **65)**	
**S0: Never severe**	95 (66)
**S1: Ever severe**	49 (34)
**Paris classification CD, *n* (%)**	226 (61.1)
**Age at diagnosis**	
**A1a: 0-<10 year**	77 (34)
**A1b: 10-<17 year**	149 (66)
**Location**	
**L1: Distal 1/3 ileal** **±** **limited cecal disease**	52 (23)
**L2: Colonic**	29 (13)
**L3: Ileocolonic**	144 (64)
**L4a: Upper disease proximal to ligament of Treitz**	40 (18)
**L4b: Upper disease distal to ligament of Treitz and proximal to distal 1/3 ileum**	11 (5)
**Behavior**	
**B1: Nonstructuring nonpenetrating**	187 (83)
**B2: Structuring**	23 (10)
**B3: Penetrating**	11 (5)
**B2-B3: Structuring and penetrating**	5 (2)
**P: Perianal disease modifier**	61 (27)
**Growth**	
**G0: No evidence of growth delay**	169 (75)
**G1: Growth delay**	57 (25)
**PUCAI at assessment, median (IQR)**	0 (0-10)
**Patients in remission, *n* (%)**	108 (75)
**wPCDAI at assessment, median (IQR)**	0 (0-10)
**Patients in remission, *n* (%)**	179 (79.2)
**Fatigue, *n* (%)**	
**No fatigue**	70 (18.9)
**Severe fatigue**	34 (9.2)
**Tanner, *n* (%)**	
**I**	44 (11.8)
**II**	48 (12.9)
**III**	67 (18.1)
**IV**	82 (22.1)
**V**	129 (34.8)
**Current treatments *n* (%)**	
**Biologics**	114 (30.8)
**Immunosuppressants**	165 (44.6)
**Corticosteroids**	22 (6)
**5-ASA**	125 (33.7)
**Other**	103 (27.8)
**Physician’s global assessment**	
**Normal**	259 (70)
**Mild**	52 (14)
**Moderate**	37 (10)
**Severe**	22 (6)

Abbreviations: CD, Crohn’s disease; IBD, inflammatory bowel disease; IBD-U, unclassified inflammatory bowel disease; IQR, interquartile range; PUCAI, Pediatric Ulcerative Colitis Activity Index; UC, ulcerative colitis; wPCDAI, weighted Pediatric Crohn’s Disease Activity Index.

No significant differences were found in the percentage of patients in remission according to the type of IBD (79.2% in Crohn’s disease [CD] vs. 75% in ulcerative colitis [UC], *P* = 0.705). However, significant differences were observed in responses to the 2 items assessing fatigue, both in the patient cohort and among their parents (Table 2).

**Table 2:** Differences in responses to the 2 fatigue-related items in both patients and their parents.

Question	No. of patients who scored 100 *n* (%)	No. of patients who scored 75 *n* (%)	No. of patients who scored 50 *n* (%)	No. of patients who scored 25 *n* (%)	No. of patients who scored 0 *n* (%)	*P*-value
**Q6: How much energy did you have during the past 2 weeks?**	97 (26.2)	186 (50.3)	53 (14.3)	28 (7.6)	6 (1.6)	0.0001
**Q32: How tired have you felt in the past 2 weeks?**	131(35.4)	118 (31.9)	56 (15.1)	46 (12.4)	19 (5.1)	
**Q6: How much energy did your child have during the past 2 weeks?**	104 (19.3)	274 (50.8)	85 (15.8)	71 (13.2)	5 (0.9)	0.0001
**Q32: How tired has your child felt in the past 2 weeks?**	195 (35.7)	158 (28.9)	107 (19.6)	59 (10.8)	27 (4.9)	

**Figure 1:**
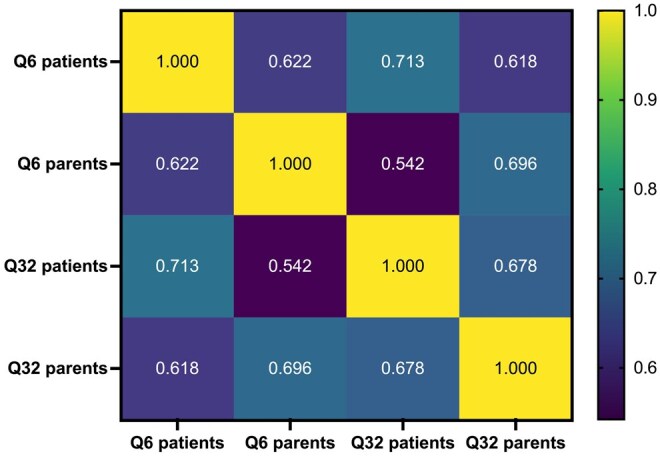
Correlation matrix among 4 fatigue-related items: Q6 and Q32 (patient responses), and Q6 and Q32 (parent responses).

Figure 1 displays the correlation matrix among 4 key variables: Q6, Q32, Q6 parents, and Q32 parents. These variables represent responses to fatigue-related items completed by both patients and their parents. All correlations were positive and statistically significant (*P* = 0.0001), indicating a consistent relationship across respondents and items. Notably, the strongest correlations were observed between patient responses to Q6 and Q32, as well as between Q32 patients and Q32 parents.

### Relationship between severe fatigue and disease activity

**Figure 2:**
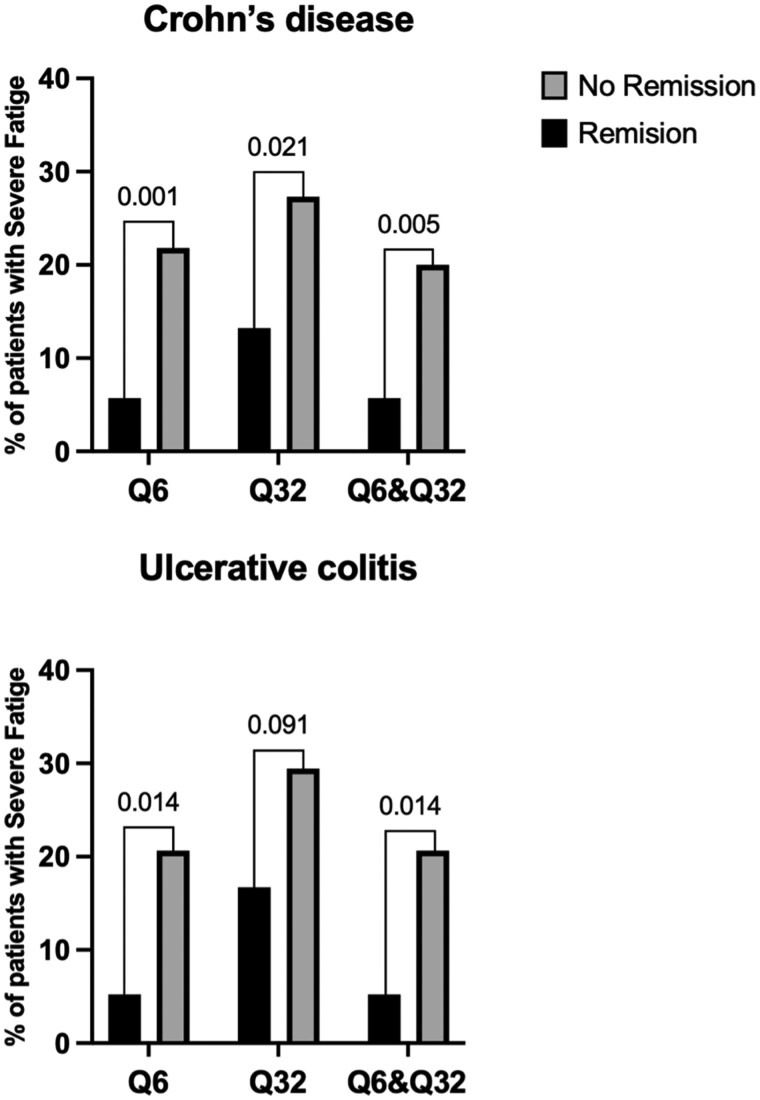
Percentage of patients with severe fatigue in relation to clinical remission (Pediatric Ulcerative Colitis Activity Index < 10 or weighted Pediatric Crohn’s Disease Activity Index ≤ 12.5). Q6: question 6 from the IMPACT-III questionnaire. Q32: question 32 from the IMPACT-III questionnaire.

Severe fatigue was significantly more prevalent among patients with active disease (Figure 2). The estimated prevalence varied depending on the IMPACT-III item used, with Q32 identifying fatigue more frequently than Q6.

### Determinants of fatigue

In the cohort of pediatric patients with IBD (Table 3), multivariate analysis identified female sex, age, and disease activity as measured by the PGA as independent predictors of severe fatigue. Dietary treatment was also associated with higher odds of severe fatigue across all 3 models (Q6, Q32, and the combined model). This association persisted after adjustment for disease duration, biological therapy, and hospitalizations.

**Table 3:** Predictive models for estimating severe fatigue.

Predictive variables of response. Multivariate analysis. Dependent variable: Severe fatigue according to Q6			
Variable	OR	95% CI	*P*
**Gender (female)**	5.436	2.223-13.288	0.0001
**Age (years)**	1.202	1.003-1.440	0.046
**Physicians global assessment (PGA)**	2.109	1.456-3.056	0.008
**Dietary treatment**	12.636	1.546-103.256	0.018

Predictive variables of response. Multivariate analysis. Dependent variable: Severe fatigue according to Q32			
**Gender (female)**	3.286	1.792-6.026	0.0001
**Age (years)**	1.245	1.087-1.427	0.002
**PGA**	1.507	1.115-2.037	0.008
**Dietary treatment**	3.340	1.205-9.257	0.02

Predictive variables of response. Multivariate analysis. Dependent variable: Severe fatigue according to Q6 and Q32			
**Gender (female)**	5.013	2.06-12.16	0.0001
**Age (years)**	1.210	1.008-1.451	0.041
**PGA**	1.945	1.342-2.819	0.0001
**Dietary treatment**	11.188	1.386-90.317	0.023

Hosmer and Lemeshow Test: *P* = 0.507. Cox-Snell *R*²: 0.116. Nagelkerke *R*²: 0.252. This table only shows the results of the variables that were finally included in the multivariate analysis. The model displayed here is significant, explains between 0.116 and 0.252 of the dependent variable, and correctly classifies 90.7% of cases. CI, confidence interval.

Hosmer and Lemeshow Test: *P* = 0.978. Cox-Snell *R*²: 0.108. Nagelkerke *R*²: 0.179. This table only shows the results of the variables that were finally included in the multivariate analysis. The model displayed here is significant, explains between 0.108 and 0.179 of the dependent variable, and correctly classifies 81.5% of cases. CI, confidence interval.

Hosmer and Lemeshow Test: *P* = 0.428. Cox-Snell *R*²: 0.104. Nagelkerke *R*²: 0.228.: This table only shows the results of the variables that were finally included in the multivariate analysis. The model displayed here is significant, explains between 0.104 and 0.228 of the dependent variable, and correctly classifies 89.5% of cases. CI, confidence interval.

In the subgroup of pediatric patients with CD (Table 4), multivariate analysis showed that female sex, presence of perianal disease, and clinical activity according to the wPCDAI index were the strongest predictors of severe fatigue. Dietary treatment also showed a consistent but more moderate association across all 3 models (Q6, Q32, and the combined model). Notably, this association persisted after adjustment for potential confounders, including disease duration, biological therapy, and hospitalizations. Perianal disease, often considered a marker of more complex disease, was also significantly associated with higher fatigue levels.

**Table 4:** Predictive models for estimating severe fatigue in patients with Crohn’s disease.

Predictive variables of response. Multivariate analysis. Dependent variable: Severe fatigue according to Q6			
Variable	OR	95% CI	*P*
**Gender (female)**	5.1	1.7-15.6	0.004
**Perianal disease**	4.1	1.3-13.1	0.015
**Active disease according to weighted Pediatric Crohn’s Disease Activity Index (wPCDAI)**	10.5	3,4-32,2	0.0001
**Dietary treatment**	3.7	1.3-10.3	0.011

Predictive variables of response. Multivariate analysis. Dependent variable: Severe fatigue according to Q32			
**Gender (female)**	4.3	1.8-9.9	0.001
**Perianal disease**	4.5	1.9-10.7	0.001
**Active disease according to wPCDAI**	4.2	1.7-10.4	0.001
**Dietary treatment**	1.9	1.2-3.1	0.006

Predictive variables of response. Multivariate analysis. Dependent variable: Severe fatigue according to Q6 and Q32			
**Gender (female)**	4.6	1.5-14.1	0.007
**Perianal disease**	4.4	1.4-13.7	0.011
**Active disease according to wPCDAI**	9.0	2.9-27.7	0.0001
**Dietary treatment**	3.5	1.3-9,6	0.015

Hosmer and Lemeshow Test: *P* = 0.256. Cox-Snell *R*²: 0.168. Nagelkerke *R*²: 0.358. This table only shows the results of the variables that were finally included in the multivariate analysis. The model displayed here is significant, explains between 0.168 and 0.358 of the dependent variable, and correctly classifies 90.4% of cases. CI, confidence interval.

Hosmer and Lemeshow Test: *P* = 0.572. Cox-Snell *R*²: 0.155. Nagelkerke *R*²: 0.260. This table only shows the results of the variables that were finally included in the multivariate analysis. The model displayed here is significant, explains between 0.155 and 0.260 of the dependent variable, and correctly classifies 82.1% of cases. CI, confidence interval.

Hosmer and Lemeshow Test: *P* = 0.412. Cox-Snell *R*²: 0.152. Nagelkerke *R*²: 0.331. This table only shows the results of the variables that were finally included in the multivariate analysis. The model displayed here is significant, explains between 0.152 and 0.331 of the dependent variable, and correctly classifies 90.4% of cases. CI, confidence interval.

In the cohort of patients with UC (Table 5), multivariate analysis identified female sex and disease activity, as assessed by the PGA, as independent predictors of severe fatigue. These associations were statistically significant across all 3 models analyzed (Q6, Q32, and the combined model). In the Q6-based and combined models, female sex showed the strongest association, with odds ratios above 8, while clinical activity also demonstrated a strong, albeit slightly lower, association. In the Q32 model, both predictors remained significant, though with more moderate effect sizes.

**Table 5:** Predictive models for estimating severe fatigue in patients with ulcerative colitis.

Predictive variables of response. Multivariate analysis. Dependent variable: Severe fatigue according to Q6			
Variable	OR	95% CI	*P*
**Gender (female)**	8.5	1.6-44.3	0.011
**Active disease according to physicians global assessment (PGA)**	6.4	1.7-24.1	0.006

Predictive variables of response. Multivariate analysis. Dependent variable: Severe fatigue according to Q32			
**Gender (female)**	3.0	1.1-8.0	0.027
**Active disease according to PGA**	3.0	3.0-1.1	0.025

Predictive variables of response. Multivariate analysis. Dependent variable: Severe fatigue according to Q6 and Q32			
**Gender (female)**	8.5	1.6-44.3	0.011
**Active disease according to PGA**	6.4	1.7-24.1	0.006

Hosmer and Lemeshow Test: *P* = 0.693. Cox-Snell *R*²: 0.104. Nagelkerke *R*²: 0.224. This table only shows the results of the variables that were finally included in the multivariate analysis. The model displayed here is significant, explains between 0.104 and 0.2224 of the dependent variable, and correctly classifies 90.5% of cases. CI, confidence interval.

Hosmer and Lemeshow Test: *P* = 0.784. Cox-Snell *R*²: 0.067. Nagelkerke *R*²: 0.106. This table only shows the results of the variables that were finally included in the multivariate analysis. The model displayed here is significant, explains between 0.108 and 0.179 of the dependent variable, and correctly classifies 80.2% of cases. CI, confidence interval.

Hosmer and Lemeshow Test: *P* = 0.693. Cox-Snell *R*²: 0.104. Nagelkerke *R*²: 0.224. This table only shows the results of the variables that were finally included in the multivariate analysis. The model displayed here is significant, explains between 0.104 and 0.2224 of the dependent variable, and correctly classifies 90.5% of cases. CI, confidence interval.

In the multivariate analysis conducted on the entire cohort, the absence of fatigue (Table 6) was significantly associated with male sex, lower pubertal stage, and not receiving treatment with biologic drugs. These 3 factors emerged as independent predictors, with male sex showing the strongest association. A lower Tanner stage was also positively associated with the absence of fatigue. Additionally, not being treated with biologics was linked to a greater likelihood of not experiencing fatigue.

**Table 6:** Predictive models for estimating absence of fatigue.

Predictive variables of response. Multivariate analysis. Dependent variable: Absence of fatigue according to the 2 questions (Q6 and Q32). Entire cohort.			
Variable	OR	95% CI	*P*
**Gender (male)**	2.5	1.3-4.6	0.003
**Lower tanner stage**	1.3	1.1-1.6	0.004
**No treatment with biologic drugs**	1.8	1.04-3.4	0.037

Hosmer and Lemeshow Test: *P* = 0.023. Cox-Snell *R*²: 0.063. Nagelkerke *R*²: 0.100. This table only shows the results of the variables that were finally included in the multivariate analysis. The model displayed here is significant, explains between 0.063 and 0.100 of the dependent variable, and correctly classifies 79.6% of cases. CI, confidence interval.

### Impact on other domains of IMPACT-III questionnaire

**Figure 3:**
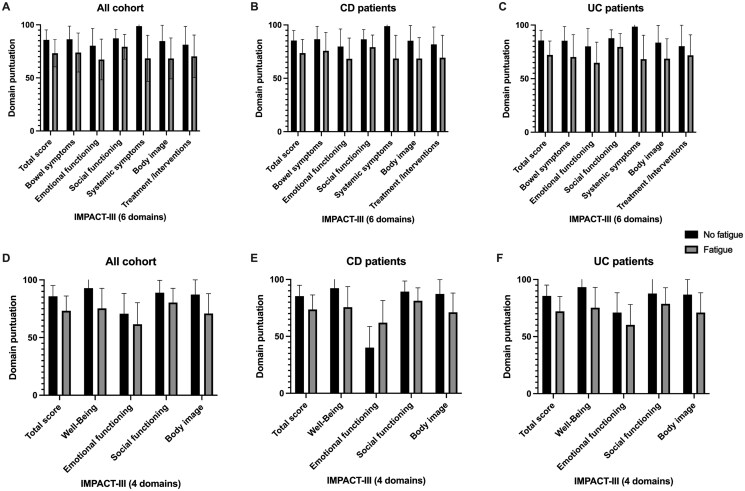
Impact of the absence of fatigue according to questions 6 and 32 across different domains. (A-C) IMPACT-III assessment with 6 domains. Questions Q6 and Q32 belong to the *Systemic Symptoms* domain. (D-F) IMPACT-III assessment with 4 domains. Questions Q6 and Q32 belong to the *Well-Being* domain.

Figure 3 shows that the presence of fatigue, regardless of its severity, is associated with a notable reduction in quality of life, as measured by the IMPACT-III questionnaire, in both the 6- and 4-domain versions. In the overall cohort, patients with fatigue had lower scores across all assessed domains, with the most pronounced differences observed in general well-being, systemic symptoms, and emotional functioning. This pattern remained consistent when analyzing by diagnostic subgroups. In CD, the impact of fatigue was particularly evident in the physical and social domains, whereas in UC, the scores were more uniformly affected. In the 4-domain model, this trend persisted, with patients with fatigue consistently showing lower global scores regardless of IBD subtype.

**Figure 4:**
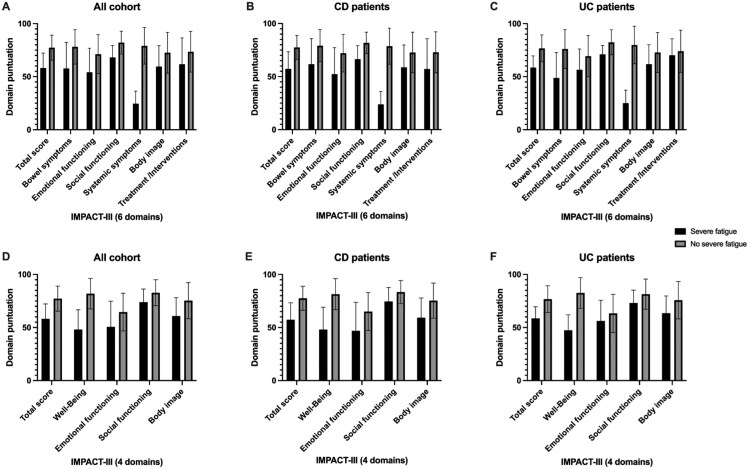
Impact of severe fatigue according to questions 6 and 32 across different domains. (A-C) IMPACT-III assessment with 6 domains. Questions Q6 and Q32 belong to the *Systemic Symptoms* domain. (D-F) IMPACT-III assessment with 4 domains. Questions Q6 and Q32 belong to the *Well-Being* domain.

Figure 4 shows that the presence of severe fatigue is associated with a significant reduction in overall quality of life scores and across all domains of the IMPACT-III questionnaire, in both the 6- and 4-domain models. This pattern was consistent in the overall cohort as well as in the analyses by diagnostic subgroup. Among patients with CD, severe fatigue was linked to greater impairment in social functioning, systemic symptoms, and general well-being. In patients with ulcerative colitis, the impact was more evenly distributed, although emotional and physical well-being were notably affected. In both disease types, patients without severe fatigue showed higher scores across all domains, highlighting the broad impact of fatigue on quality of life regardless of IBD subtype.

## Discussion

Fatigue emerged as a highly prevalent symptom in our cohort, with 81.1% of pediatric patients with IBD reporting some degree of fatigue at the time of evaluation. This figure is in line with previous reports, such as the study by Turner et al.,^6^ which identified fatigue in 78% of patients at diagnosis and in over 30% 1 year later, irrespective of disease activity.

A variety of tools have been employed to assess fatigue in pediatric IBD, though few are disease-specific or validated for this age group. Among the most commonly used are the Peds FACIT-F,^7^ initially developed for children with cancer; the PedsQL multidimensional fatigue scale (MDFS), a generic but age-adapted and proxy-report tool;^2^^,^^8–10^ the IMPACT-III,^6^^,^^11^^,^^12^ an IBD-specific quality of life questionnaire that includes items on fatigue; and the TUMMY-UC, a scale targeting pediatric ulcerative colitis that includes an item on weakness.^13^ Other instruments include the PROMIS Pediatric v2.0, which assesses physical, mental, and social health in chronic conditions,^12^^,^^14^^,^^15^ and the KIDSCREEN-27, which, despite being a general quality of life instrument, captures fatigue-related aspects.^11^ Some instruments validated in adults have also been used to assess fatigue in children with IBD, including the multidimensional fatigue inventory (MFI),^16^ the multidimensional assessment of fatigue (MAF),^17^ and the IBD-F.^18^ In our study, we utilized the IMPACT-III questionnaire alongside its parent-reported version, both of which were translated and culturally adapted into Spanish by our group.^5^

Interestingly, when comparing patient and parent reports on fatigue-related items in IMPACT-III, we found a significant discrepancy in perceived energy levels (item Q6), with patients reporting higher energy more frequently than their parents. However, no significant difference was found in the item that directly assessed tiredness (item Q32). These results suggest that while both patients and caregivers may perceive fatigue similarly, their interpretation of “energy” may differ. We hypothesize that this divergence stems from semantic and sociocognitive factors: the term “fatigue” is concrete and emotionally salient, whereas “energy” is a more abstract and socially loaded concept, possibly leading to underreporting. Furthermore, admitting low energy may carry greater stigma or be associated with more severe illness, reducing its perceived acceptability as a response.

In our opinion, question 32 (“How tired have you felt in the past 2 weeks?”) appears to detect fatigue more frequently than question 6 (“How much energy did you have during the past 2 weeks?”), likely due to several semantic, psychometric, and sociocognitive factors. First, question 32 explicitly refers to the symptom of tiredness using language that is concrete, emotionally salient, and commonly used by patients. In contrast, question 6 requires a positive appraisal of one’s level of energy, which is a more abstract and functionally oriented construct. This distinction may lead to more diffuse or conservative responses in question 6. Second, individuals are generally more inclined to acknowledge feeling tired than to report having little or no energy, as the latter may be perceived as a sign of more severe health deterioration. This perception bias may result in underreporting of fatigue in responses to question 6. Third, reporting tiredness tends to carry less social stigma than admitting to a lack of energy, which can be associated with weakness, depression, or disability, making question 32 more permissive for the expression of symptoms. Lastly, the response threshold may differ between items: responses such as “somewhat tired” or “quite tired” in question 32 are often sufficient to classify the presence of fatigue, whereas a response like “some energy” in question 6 may not be interpreted as clinically relevant fatigue, depending on the criteria applied. Taken together, these differences in symptom framing, response processing, and social desirability likely contribute to the greater sensitivity of question 32 in detecting fatigue, albeit potentially at the cost of reduced specificity.

Most pediatric studies have reported a significant association between fatigue and disease activity; however, a considerable proportion of patients in clinical remission continue to experience fatigue. In our cohort, 287 out of 350 (77.5%) patients were in clinical remission, yet only 18.9% reported no fatigue at all. This percentage was even higher than that observed in the series by Turner et al.,^6^ in which 63% of patients in clinical remission ­reported some degree of fatigue. A prospective cohort study evaluating 326 patients with IBD initiating biologic therapy found that, although fatigue improved in parallel with clinical response, a substantial proportion of patients continued to experience fatigue despite achieving remission. At baseline, 61% reported significant fatigue, which was associated with female sex, depressive symptoms, active disease, and poor sleep quality. While clinical remission reduced the likelihood of persistent fatigue at all time points, up to 28% of patients in remission remained fatigued at 1 year, underscoring that fatigue in IBD is a multifactorial symptom not solely explained by inflammatory activity.^19^ A prospective study evaluating the relationship between IGF-1, proinflammatory cytokines, and fatigue in children with IBD demonstrated that even in the absence of clinically active disease, patients reported significantly lower generic quality of life and greater fatigue compared to healthy peers. Notably, those with IGF-1 z scores in the lowest quartile exhibited more fatigue and poorer disease-specific HRQOL, alongside elevated levels of interleukin (IL)-6, IL-10, IL-17A, and interferon-γ. These findings suggest that fatigue in pediatric IBD may, in part, be mediated by a cytokine-driven mechanism involving growth hormone resistance, highlighting a potential pathophysiological link between chronic inflammation, altered growth signaling, and persistent fatigue.^20^

No significant association has been found between anemia and fatigue.^6^^,^^8^ However, slower improvement in fatigue has been linked to hypophosphatemia, particularly following administration of ferric carboxymaltose compared to ferric derisomaltose (51% vs. 8%, respectively).^21^ These findings support the rationale for monitoring serum phosphate levels after intravenous iron administration.

When considering factors beyond disease-related variables and activity, our study found that the absence of fatigue was independently associated with male sex, earlier pubertal stage, and not receiving biologic therapy. Among these, male sex showed the strongest association, suggesting a potential influence of sex-related biological or psychosocial factors on the perception of fatigue. The association with lower Tanner stage may indicate that pubertal development affects how symptoms such as fatigue are experienced or reported. Interestingly, not being treated with biologics was also linked to a higher likelihood of being fatigue-free, which may reflect a milder disease course or a lower perception of illness among patients not receiving advanced therapies. In this regard, a randomized controlled trial assessing the impact of sleep interventions in children and adolescents with IBD in clinical remission found that both sleep tracking and in-person sleep hygiene counseling led to ­significant improvements in sleep/rest fatigue scores, as measured by the PedsQL-MDFS. In particular, in-person counseling was associated with beneficial behavioral changes, such as increased avoidance of electronic screens before bedtime. These findings suggest that simple, low-cost interventions targeting sleep hygiene may effectively reduce fatigue and improve sleep-related outcomes in pediatric IBD patients.^2^

Taken together, our results and those from previous literature underscore the complexity of fatigue in pediatric IBD. It is a multifactorial symptom influenced by inflammation, growth signaling, psychosocial context, and behavioral factors. Despite its high prevalence and impact on quality of life, fatigue remains under-recognized and poorly assessed in clinical practice. There is a clear need to develop and validate disease-specific instruments tailored to pediatric populations to improve the evaluation, monitoring, and management of fatigue in children with IBD.

This study has several limitations that should be acknowledged. Fatigue was assessed using items from the IMPACT-III questionnaire, which is not a validated tool for fatigue measurement and lacks standardized cut-off points. Although this approach allowed us to capture fatigue perception in a large multicenter cohort, the absence of formal validation restricts the precision of our estimates. Therefore, our findings on the prevalence and predictors of fatigue should be interpreted with caution, and future studies should employ validated pediatric fatigue-specific instruments to confirm and expand these results. Moreover, although we adjusted for key confounders, residual confounding cannot be excluded, ­particularly regarding treatment variables. Finally, the cross-sectional design precludes causal inferences between fatigue, disease activity, and treatment exposure. While our findings highlight relevant associations, they cannot establish directionality or causality; longitudinal studies are warranted to clarify the temporal relationship between fatigue and the disease course, as well as the potential impact of therapeutic strategies on fatigue outcomes.

## Conclusions

Fatigue is increasingly recognized as a highly prevalent and burdensome symptom in children with IBD. While fatigue is frequently associated with active disease and specific phenotypic features such as extensive intestinal involvement or the need for immunosuppressive therapy, our findings and those of previous studies demonstrate that a substantial proportion of patients continue to experience fatigue even during periods of clinical remission. This persistence suggests that fatigue in IBD is not solely driven by inflammation, but rather arises from a complex interplay of physiological, psychological, and behavioral factors.

Accordingly, the comprehensive assessment of fatigue should include a systematic search for modifiable or treatable contributors, including anemia, micronutrient deficiencies (eg, iron, phosphate), poor sleep quality, psychosocial distress, and the side effects of pharmacological treatments. Despite its high prevalence and clinical relevance, fatigue is often under-recognized and insufficiently evaluated in pediatric IBD. One major limitation in current practice is the lack of a validated, disease-specific instrument tailored to assess fatigue in this age group. Existing tools are either generic or adapted from adult populations, limiting their sensitivity to capture the unique experiences and developmental context of children and adolescents with IBD.

Therefore, there is a clear and urgent need to develop and validate a standardized, age-appropriate, and disease-specific questionnaire that allows for the accurate assessment of fatigue in pediatric IBD. Such a tool would not only enhance clinical evaluation and individualized care but also facilitate future research aimed at understanding the pathophysiology, natural history, and therapeutic targets of fatigue in this population.

## Data Availability

The datasets generated and/or analyzed during the current study are not publicly available due to patient privacy and ethical restrictions. However, they are available from the corresponding author upon reasonable request and subject to appropriate justification and institutional approval.
